# Orthorexia nervosa: An integrative literature review of a lifestyle syndrome

**DOI:** 10.3402/qhw.v10.26799

**Published:** 2015-08-14

**Authors:** Linn Håman, Natalie Barker-Ruchti, Göran Patriksson, Eva-Carin Lindgren

**Affiliations:** 1 Department of Food and Nutrition and Sport Science, University of Gothenburg, Gothenburg, Sweden; 2 School of Health and Welfare, Halmstad University, Halmstad, Sweden

**Keywords:** Disordered eating, excessive exercise, food regulation, health, healthism, sport

## Abstract

Bratman first proposed orthorexia nervosa in the late 1990s, defining it an obsession with eating healthy food to achieve, for instance, improved health. Today, in the Swedish media, excessive exercising plays a central role in relation to orthorexia. A few review articles on orthorexia have been conducted; however, these have not focused on aspects of food and eating, sport, exercise, or a societal perspective. The overall aim of this study was to provide an overview and synthesis of what philosophies of science approaches form the current academic framework of orthorexia. Key questions were: What aspects of food and eating are related to orthorexia? What role do exercise and sports play in relation to orthorexia? In what ways are orthorexia contextualized? Consequently, the concept of healthism was used to discuss and contextualize orthorexia. The method used was an integrative literature review; the material covered 19 empirical and theoretical articles published in peer-reviewed journals. This review demonstrates a multifaceted nature of orthorexia research; this field has been examined from four different philosophies of science approaches (i.e., empirical-atomistic, empirical-atomistic with elements of empirical-holistic, empirical-holistic, and rational-holistic) on individual, social, and societal levels. The majority of the articles followed an empirical-atomistic approach that focused on orthorexia as an individual issue, which was discussed using healthism. Our analysis indicates a need for (a) more empirical-holistic research that applies interpretive qualitative methods and uses a social perspective of health, e.g., healthism and (b) examining the role of sports and exercise in relation to orthorexia that takes the problematizing of “orthorexic behaviours” within the sports context into account.

In the end it was impossible to exercise more or eat less. It was impossible to vomit more or feel worse. Everything was anxiety. I was unable to exercise to remove the anxiety. I had terrible pain and felt like shit. At the same time, I felt that if I was not exercising, I would die. Then I would have to kill myself. (von Wachenfeldt, 2010)

This personal account by an individual who was considered to have orthorexia was printed in a Swedish newspaper. The article focused on the individual's pain and suffering due to orthorexia nervosa. Orthorexia nervosa was termed in the late 1990s by Bratman (1997) in a non-scientific journal. Bratman (Bratman & Knight, 2000, p. 9) defined orthorexia as “a fixation on eating healthy food” in order to avoid ill health and disease. Orthorexia is described as often starting innocently with a desire, for instance, to improve one's diet and/or eating habits or general health (Bratman & Knight, 2000). Currently, orthorexia is not recognized as a disease by the fifth edition of the Diagnostic and Statistical Manual of Mental Disorders (Varga, Dukay-Szabó, Túry, & Van Furth, 2013). Physicians and scholars alike have questioned whether orthorexia should be considered a disorder (Rössner, 2004), a behavioural addiction (Marazziti, Presta, Baroni, Silvestri, & Dell'Osso, 2014), or an extreme dietary habit (Varga et al., 2013). In addition, orthorexia has also received attention in the Swedish media that similarly highlights the condition's fanatic eating habits, but in contrast also depicts excessive exercise to play a significant role in relation to orthorexia.

Since Bratman's initial work in 1997, orthorexia has consequently gained attention in both the media (Vanderycken, 2011) and international research (e.g., Varga et al., 2013). Less than a decade after the term was introduced, the first scientific article addressing orthorexia was published (Donini, Marsili, Graziani, Imbriale, & Cannella, 2004). Although research related to orthorexia is still ongoing (Vanderycken, 2011), orthorexia can be considered to be a new and emerging topic. During such times of conceptualization, integrative reviews have been proposed as useful because they contribute to the understanding of a topic (e.g., Torraco, 2005). Reviews also provide an overview of existing knowledge and thus identify gaps, inconsistencies, and future research directions that should be developed or negotiated (cf. Stambulova & Ryba, 2014). To the best of our knowledge, three review articles have explicitly focused on orthorexia (Brytek-Matera, 2012; Chaki, Pal, & Bandyopadhyay, 2013; Varga et al., 2013). These articles usefully summarize cross-sectional studies and case studies. For instance, background factors, individual characteristics, assessment instruments, overlap and relation with other disorders, and comorbidities are examined in relation to orthorexia (Brytek-Matera, 2012; Chaki, Pal, & Bandyopadhyay, 2013; Varga et al., 2013). To date, these findings are disparate and no clear picture regarding orthorexia can be presented. However, in line with Bratman's description of orthorexia, all these reviews are based on the notion that obsession with food and eating is one of the main elements of orthorexia. Even so, various content-related aspects of food and eating have not been in focus within previous reviews. Because food and eating can be regarded as main elements of orthorexia, it is of interest to explore these aspects as they contribute to define orthorexia. However, given that excessive exercise plays a significant role in relation to orthorexia within the Swedish media, this relation ought to be explored in this review study to gain an overview of what the research shows related to this. Sports and exercise in relation to orthorexia have not been addressed sufficiently in previous review studies. Prior reviews essentially focused on statistical research and excluded existing research conducted from a qualitative perspective and using social theories. The overall aim of this study was to provide an overview and synthesis of what philosophies of science approaches form the current academic framework of orthorexia. Key questions were: (a) What aspects of food and eating are related to orthorexia? (b) What role do exercise and sports play in relation to orthorexia? (c) In what ways is orthorexia contextualized? In order to contextualize the orthorexia research included in this integrative review, the theoretical concept of healthism (Crawford, 1980) was used.

## Healthism: *a* social perspective of health

Healthism refers to a social construction of health (cf. Lee & Macdonald, 2010). This view of health is described as a dominant ideology within contemporary developed Western societies and can be observed in health promotion efforts, popular media (Lee & Macdonald, 2010), and advertising (Dworkin & Wachs, 2009; Kirk & Colquhoun, 1989). The concept of healthism was termed by Crawford (1980) when he was describing the “new health consciousness” that emerged during the 1970s. During this period, society placed more emphasis on individual responsibility for health, which meant that health problems and their solutions became situated at the level of the individual in contrast to previous state directions. Because of the emphasis on the individual, these movements can be understood as political (Crawford, 2006; Kirk & Colquhoun, 1989). A healthiest view of health (i.e., healthiest is the term of health within healthism) assumes that health can be achieved with relative ease with a focus on body size and shape through individual discipline and moral conduct (Crawford, 1980).

Healthism's emphasis on personal responsibility demands that individuals achieve health, avoid risk factors, and prevent ill health (Crawford, 1980). Actions for achieving health are typically regarded as regular exercise (Kirk & Colquhoun, 1989) and healthy eating habits (Wright, O'Flynn, & Macdonald, 2006). Another element of healthism is moral obligation, which is linked to body size and shape (Crawford, 1980). Healthism considers an attractive and youthful body, along with healthy eating and regular exercise, to be an element and marker of good health (cf. Crawford, 1987). Healthism can also be linked to discourses of contemporary consumer culture (Wright et al., 2006). Within such cultures, a fit body represents health and is also a symbol of good living; an obese body represents laziness, emotional weakness, and unattractiveness (Crawford, 1987).

Given the emphasis on individual responsibility and moral obligation, healthism shapes numerous behaviours and practices surrounding health, exercise, and eating. In turn, these practices can contribute to both improving one's habits and to promoting unhealthy notions about the body, exercise, and diet (Lee & Macdonald, 2010) including constrained behaviours or self-surveillance in response to normative social pressures to achieve health and to reduce perceived health risks (Crawford, 2004). Even detrimental behaviours or destructive efforts can be adopted to conform to ideals and to live healthfully (cf. Rich & Evans, 2009). Dieting and exercise are, therefore, markers not only of improved health but also of enhanced security (Crawford, 2004). Healthism shapes individuals’ perceptions of health and attractive bodies (Barker-Ruchti, Barker, Sattler, Gerber, & Pühse, 2013). Attempts to live healthily and perceptions of health are thus not merely understood on an individual level. The social perspective of health also shapes individuals. This study, therefore, contextualizes and discusses orthorexia using the concept of healthism.

## Methods

### An integrative review

In order to include and combine theoretical and empirical literature with a wide range of aims and concept definitions, this study was designed as an integrative review (see Torraco, 2005; Whittemore & Knafl, 2005). An integrative review differs from other forms of literature reviews in that the former enables a broader inclusion of data (e.g., quantitative and qualitative research as well as theoretical and methodological literature) and the latter largely examines studies that apply similar methodologies (Whittemore, 2005).

### Materials and data collection

**Figure 1:**
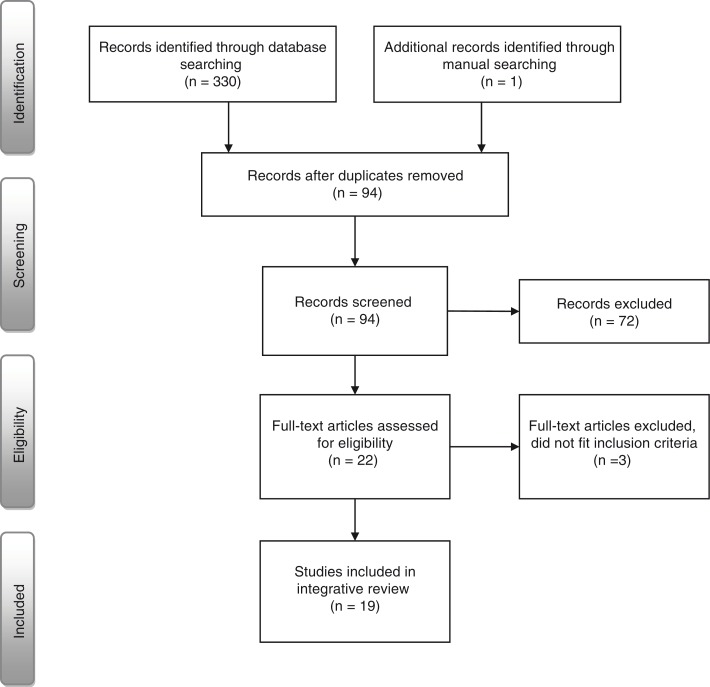
Flow of information through the different phases of the integrative review (Moher et al., 2009).

A sample of international scientific articles has been collected. The inclusion criteria were articles that dealt explicitly with orthorexia, were published in internationally peer-reviewed journals written in English, and included either empirical or theoretical content. The exclusion criteria were articles that exclusively focused on instruments (e.g., construction and/or development). In June 2014, a systematic literature search using the databases Academic Search Elite, PubMed, Science Direct, SPORTDiscus, and Summon was conducted. The keywords used were orthorexia, orthorexia nervosa, and orthorexic society. The PRISMA flow diagram (outlined in Figure 1) has been used to illustrate the identification, screening, eligibility, and inclusion processes (Moher, Liberati, Tetzlaff, Altman, & The PRISMA Group, 2009). In total, 330 articles (including duplicates) published between January 2004 and June 2014 were collected. A manual search was also conducted and one additional article (Nicolosi, 2006) was included from the reference list. The manual search involved checking the reference lists of the collected articles: previous review articles that solely focused on orthorexia (Brytek-Matera, 2012; Chaki, Pal, & Bandyopadhyay, 2013; Varga et al., 2013); review articles that included orthorexia in part, for instance, along with eating disorders (Babicz-Zielińska, Wądołowska, & Tomaszewski, 2013); and behavioural addictions (Marazziti et al., 2014), doctoral theses (McInerney-Ernst, 2011), and master's theses (Shah, 2012). The reference list in one doctoral thesis (Borgida, 2011) was not checked because this document could not be accessed. After this identification process, 237 duplicates were removed. Ninety-four records (titles and abstracts) were screened to determine their adherence to the inclusion criteria and research questions. Seventy-two publications did not fulfil the inclusion criteria or respond to the research questions. The remaining 22 full-length articles were then again assessed for eligibility in light of the inclusion criteria. Three articles were excluded because they did not fit inclusion criteria. Finally, 19 peer-reviewed empirical and theoretical articles (15 empirical articles, 1 theoretical article, and 3 case reports) were included in this integrative review.

### Analytical procedure

**Figure 2:**
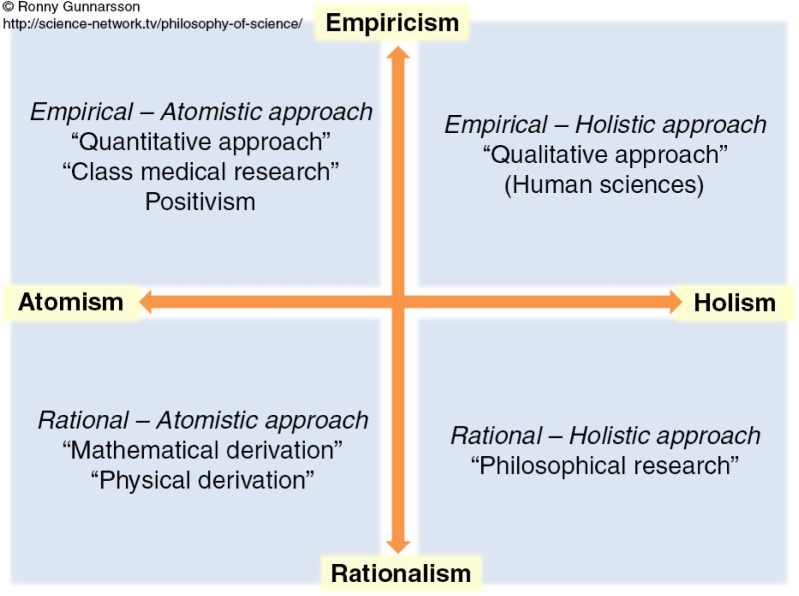
Philosophy of science world map (Gunnarsson, 2014). Reproduced with permission from Gunnarsson.

The articles were initially read to obtain an overall picture of their content. Next, the articles’ contents were compiled into a table including author and year of publication; objectives, participants, and design; and type of orthorexia examined (see Table I^1^ for an overview of the included research articles presented according to design in alphabetical order by author). A summary of the findings was also written. This documentation revealed a disparate picture among objectives, participants, type of orthorexia examined, and findings. The philosophy upon which a study's scientific assumptions are based affects how orthorexia is researched because the philosophy shapes the research aims, research questions, data collection or production methods, and analytic procedures (Gunnarsson, 2014). In order to identify and sort which scientific approaches the articles had adopted, Gunnarsson's (2014) philosophy of science world map was used (Figure 2). Gunnarsson's (2014) map contains four different approaches: empirical-atomistic, empirical-holistic, rational-holistic, and rational-atomistic. This map allows scholars to understand the different approaches without getting distracted by too many details. The articles were sorted based on Gunnarsson's (2014) map in the following steps: (a) each article's objectives, the type of orthorexia examined, and the findings were compared with the four different approaches outlined in Figure 2; and (b) the articles were sorted along the different approaches outlined by Gunnarsson's (2014) map.

**Table I:** An overview of included articles; presented according to design in alphabetical order by author.

Author and year of publication	Objectives	Participants	Design	Type of orthorexia examined
Aksoydan and Camci (2009)	Determine the prevalence of orthorexia nervosa and other associated factors	94 performance artists; opera singers, ballet dancers, and symphony orchestra musicians	Cross-sectional study	Interviewed ORTO-15 test
Arata, Battini, Chiorri, and Masini (2010)	Investigate prototypical patterns of eating habits and behavioural and psychological correlates	1388 students from public middle and high schools	Cross-sectional study	ORTO-15 test
Arusoğlu, Kabakçi, Köksal, and Merdol (2008)	Reveal the psychometric properties of the Turkish version of ORTO-15, and to investigate the relationship between orthorexia, and eating attitude, obsessive-compulsive symptoms, and some demographic variables	994 academic and administrative personnel from a University	Cross-sectional study	ORTO-15/11 test
Bağci Bosi, Çamur, and Güler (2007)	Investigate prevalence of orthorexia nervosa or highly sensitive attitudes in the eating behaviour, and to examine the effect of certain factors on eating habits	318 resident medical doctors working in a Faculty of Medicine	Cross-sectional study	ORTO-15 test
Brytek-Matera, Krupa, Poggiogalle, and Donini (2014)	Validate the Polish version of the ORTO-15 test	400 University students, administrative and teaching personnel	Cross-sectional study	ORTO-15 test
Donini et al. (2004)	A tentative proposal for the diagnosis of orthorexia and the verification of its prevalence	404 subjects with various different occupational characteristics	Cross-sectional study	“Health fanatic” eating habits and Scale 7 of the Minnesota Multiphasic Personality Inventory
Eriksson, Baigi, Marklund, and Lindgren (2008)	Investigates how scores on the Social Physique Anxiety Scale and the Sociocultural Attitudes Towards Appearance Questionnaire relate to Bratman's orthorexia test scores with regard to age, sex, and self-reported exercise frequency and duration	251 participants in fitness centre activities	Cross-sectional study	The 10-question Bratman test for orthorexia (BOT)
Fidan, Ertekin, Işikay, and Kirpinar (2010)	Determine the prevalence of orthorexia and to examine the effect(s) of some socioeconomic factors on eating habits	878 medical students	Cross-sectional study	ORTO-11 test
Koven and Senbonmatsu (2013)	Determine whether orthorexic individuals experience the same cognitive problems using standardized neuropsychological tests sensitive to performance differences in these domains	100 right-handed young adults from undergraduate college courses	Cross-sectional study	ORTO-15 test
Tomsa, Istfan, Jenaro, Flores, Belén, and Bermejoc (2012)	Verify the psychometric properties of the Body Image Screening Questionnaire for Eating Disorder Early Detection	156 clinical and general populations	Cross-sectional study	Body Image Screening Questionnaire—all subscales; Bulimia; Anorexia; Orthorexia; Perception of Obesity; Vigorexia
Valera, Ruiz, Valdespino, and Visioli (2014)	Examine prevalence of orthorexia nervosa	136 local ashtanga yoga practitioners	Cross-sectional study	ORTO-15 test
Varga, Konkolÿ-Thege, Dukay-Szabó, Túry, and Van Furth (2014)	Examine the psychometric properties of a Hungarian adaptation (ORTO-11-Hu), and to investigate its relationship to food consumption and lifestyle habits in order to contribute to a better description of the phenomenon	810 participants; students, graduated healthcare professionals, and non-healthcare professionals	Cross-sectional study	ORTO-11-Hu test (modified ORTO 15)
Korinth, Schiess, and Westenhoefer (2009)	Examine: (a) whether students of nutrition differ from other students in the extent of disordered eating patterns; (b) whether the extent of such disordered eating patterns changes during the course of the study programme; and (c) whether students of nutrition improve healthy food choices in parallel to their increasing knowledge of nutrition	219 students of nutrition or nutrition and home economics. 123 freshmen and 96 seventh semester or higher. The control group: 114 students. 68 students were freshmen and 46 were from the seventh semester or higher	Cross-sectional comparison study	The 10-question Bratman Test for orthorexia (BOT)
Segura-García et al. (2012)	Occurrence of orthorexia nervosa in athletes and to verify the relationship between orthorexia nervosa and eating disorders	577 athletes (taekwondo, boxing, judo, body building, volleyball, basketball, soccer, aerobics and aqua fitness). 217 sedentary matched controls	Cross-sectional comparison study	ORTO-15 test
Moroze, Dunn, Holland, Yager, and Weintraub (2014)	Report of a patient with disordered eating closely resembles the descriptions for orthorexia	A 28-year-old male	Case report	Clinical case
Park et al. (2011)	Report of a patient with orthorexia nervosa who developed serious metabolic conditions	A 30-year-old male	Case report	Clinical case
Zamora, Bonaechea, Sánchez, and Rial (2005)	A clinical case that responds to the characteristics of orthorexia nervosa. The differential diagnosis with chronic delusional disorder, anorexia nervosa, and obsessive–compulsive disorder is carried out	A 28-year-old woman	Case report	Medical record
Rangel, Dukeshire, and MacDonald (2012)	Explore what factors influence the dietary behaviour of women according to different life stages (e.g., adolescent living home, young adult living alone, living with partner without children and parenthood)	52 women at four different life stages	Explorative (a grounded theory approach)	Focus groups interviews
Nicolosi (2006)	Interpreting current alimentary anxieties and the widespread hostility towards biotechnologies by defining contemporary society as an orthorexic society	—	Theoretical study	Theories by Fischler and Falk

The philosophy of science world map builds on various ontological, epistemological, and methodological assumptions and approaches to yield an overview of these approaches. The horizontal arrow represents the ontological assumptions of atomism versus holism. Atomism refers to a view in which the world is seen as the sum of the parts; holism refers to the world being larger than the sum of its parts (Gunnarsson, 2014). Thus, an atomistic view of orthorexia would explore it as a sum of individuals and their perceptions, intentions, and actions; a holistic view would consider sociocultural aspects surrounding orthorexia. The vertical arrow represents two epistemological assumptions: empiricism versus rationalism (Gunnarsson, 2014). Empiricism refers to experience (observing) and rationalism refers to reasoning (i.e., logical assumptions) as a foundation for knowledge (Gunnarsson, 2014; Lewis-Beck, Bryman, & Liao, 2007). In combination, the arrows create four orientations that represent major methodological approaches that can be followed when knowledge is produced. The first methodological approach, “empirical-atomistic,” builds on empiricism and atomism is characterized by the “quantitative approach” and a “positivistic and classic medical focus.” The “empirical-holistic” approach involves empiricism and holism and reflects a “qualitative approach” and “human scientific focus.” The “rational-holistic” approach refers to rationalism and holism and is philosophically driven. Finally, the “rational-atomistic” approach builds on rationalism and atomism and comprises mathematical and physical derivations.

In addition to Gunnarsson's (2014) philosophy of science world map, data were extracted to summarize and code the articles’ contents (i.e., research aims, designs, findings, background variables that pertain to orthorexia, and theoretical framework). This explorative process included the following steps: First, the codes were compared to extract similarities and differences and to create tentative categories. Then, the tentative categories were reviewed and discussed several times and then revised and encoded into six categories. Finally, these categories were sorted and grouped to identify the different approaches outlined in Figure 2. The analytical procedure may look like a linear process but it actually involved a back-and-forth process between different parts and the whole. In a final analytic step, the different approaches and categories were sorted according to an individual, social, and societal level to synthesize the findings within his study (cf. levels within social-ecological model; Stokols, 1996).

## Findings

The analysis revealed that four approaches formed the current academic frameworks of orthorexia based on Gunnarsson's (2014) philosophy of science world map: (a) empirical-atomistic, (b) empirical-atomistic with elements of empirical-holistic, (c) empirical-holistic, and (d) rational-holistic. One mixed approach was added to Gunnarsson's (2014) map to reflect multiple approaches because some of the articles’ contents belonged to the empirical-atomistic approach but contained elements of the empirical-holistic approach. There were, for instance, articles that researched the prevalence of orthorexia and yet discussed environmental influences throughout the article. The six derived categories were divided accordingly into the approaches discussed above as follows: two categories within the first approach, two categories within the second approach, one category within the third approach, and one category within the fourth approach. Each category within the different approaches is presented below.

### Empirical-atomistic approach

This approach deals with findings within case studies and high scores on orthorexia instruments^2^ in relation to the categories *food choices*, *approach and regulation*, and *perception of and relation to the body*. Various questionnaires have been used to measure the prevalence of orthorexia. Two of these questionnaire will be discussed here: Bratman's orthorexia test (BOT) and the ORTO-15 test, plus a short version of ORTO-15, called the ORTO-11 test, wherein four items were removed in order that the test include only items that were statistically powerful (Arusoğlu et al., 2008). The BOT questions were created by Bratman (Bratman & Knight, 2000) and some of the questions in ORTO-15 were developed from BOT.

As discussed above, different instruments have been used to examine the “prevalence” of orthorexia among different groups. The reported “prevalence” ranges from 6.9% among individuals with various occupational characteristics in Italy (Donini et al., 2004) to 86% among ashtanga yoga practitioners in Spain (Valera et al., 2014). This approach encompasses the two categories presented in italics below.

The categories *food choices* and *approach and regulation* include articles that link higher scores on the orthorexia instruments to different aspects of food and eating. In general, higher scores are related to abnormal eating habits (Brytek-Matera et al., 2014, Segura-García et al., 2012) and a pathological or distorted eating attitude (Arusoğlu et al., 2008, Fidan et al., 2010). More specifically, higher scores are linked to nutritional quality of food (Bagci Bosi, Çamur, & Güler, 2007; Donini et al., 2004) such as homemade or organically produced food (Donini et al., 2004). A similar emphasis on quality of food is supported by a case report (Moroze et al., 2014). In addition, individuals who scored higher avoided certain foods (Segura-García et al., 2012), did not consume alcohol (Aksoydan & Camci, 2009, Varga et al., 2014), avoided foods with specific colours, consumed more healthy foods (e.g., whole wheat cereals, fruit and vegetables; Varga et al., 2014), and regarded preserved food as dangerous (Donini et al., 2004). Similarly, individuals with more unhealthy eating habits generally scored lower (Arata et al., 2010). In addition, higher scores are also linked to (a) replacing lunch or dinner with salad or fruit, (b) looking at the contents of the food that is bought, and (c) reporting that the content of the product was important in the decision to purchase it (Bagci Bosi et al., 2007). Within a clinical case, it has been noted that obsessive ideas about food were based on personal given criteria for what is considered to be healthy (Zamora et al., 2005).

Other aspects that were related to higher scores were a fear of eating in front of other people (Segura-García et al., 2012) and a strong or uncontrollable need to eat, composed with feelings of guilt or nervousness (Donini et al., 2004).

Food choices and approach and regulation also include that individuals who score high on orthorexia instruments (a) follow a fixed schedule, (b) spend a significant amount of time preparing meals, and (c) tend to eat the same foods every day (Varga et al., 2014). A case study also noticed that the same food and minimal amounts of food was ingested every day (Park et al., 2011). Furthermore, high scores were also linked to special diets (Varga et al., 2014), vegetarian diets (Valera et al., 2014), previous dieting (Segura-García et al., 2012), and repeated attempts to diet (Tomsa et al., 2012). In addition, restricted eating habits and food choices were also noticed within a case study. These habits and choices were based on raw food vegan diet, vegetarian, or spiritual ideologies (Zamora et al., 2005).

The second category concerns *perception of and relation to the body*. One study indicated that men's internalization of a muscular body ideal was related to higher scores. For women, the results indicated that exercise frequency, social physique anxiety (SPAS; concerns about body appearance in social settings), awareness, and internalization of a thin body are ideally related to higher scores (Eriksson et al., 2008).

Body weight concerns are also related to high scores (Brytek-Matera et al., 2014). One study found that large changes in weight over a short time period were experienced by individuals who scored high on these instruments (Tomsa et al., 2012). Moreover, individuals who scored higher considered being overweight to be a sign of weakness and blamed the victims for their diseases (Varga et al., 2014). Another way in which the body was ascribed meaning is visible within a case study, “treating my body like a temple and giving it the pure building blocks it needs” (Moroze et al., 2014, p. 7). However, in the same case report, neither preoccupation with appearance nor interest in preventing weight gain was reported. Similarly, another case report did not detect obsessive ideas regarding weight, anxiety about gaining weight, distortion of the body image, the desire to be thin, or anxiety regarding progressive weight gain (Zamora et al., 2005).

### Empirical-atomistic approach with elements of the empirical-holistic approach

This approach deals with empirical-atomistic research that includes environmental and social aspects. Two categories belong to this approach and are presented in italics below.

Within the *education area*, Korinth et al. (2010) found that there was no difference in the average disinhibition scores during the first two semesters between nutrition students and students of other majors who served as controls. The number of controls who scored higher on the orthorexia instrument remained the same over time, whereas it decreased for the nutrition students between the first and second semesters and subsequent semesters (Korinth et al., 2010).

This category refers to the influence of *cultures of professional and leisure activities*, which is discussed in relation to higher scores on orthorexia instruments. Performance artists (i.e., opera singers, ballet dancers, and symphony orchestra musicians) scored high in general (Aksoydan & Camci, 2009). Aksoydan and Camci (2009) argue that these individuals might have scored higher because of their higher socioeconomic level and higher level of education than the majority of the general public. In addition, artists are role models for the general public in terms of physical appearance and lifestyle. The ballet dancers scored the lowest among the performance artists. This fact might be because of the definition of orthorexia not being consistent with their eating habits and lifestyles; ballet dancers are more likely to suffer from anorexia and bulimia. In another study, the high scores on orthorexia instruments among ashtanga yoga practitioners were discussed in terms of the culture within yoga activities because these activities often require self-discipline lifestyle modifications such as healthier eating habits (Valera et al., 2014). Thus, Valera et al. (2014) stress that higher scores do not necessarily indicate destructive behaviours.

A relation between sports/exercise and higher scores on orthorexia instruments was revealed. One study found that exercise frequency, along with SPAS scores and sociocultural attitudes towards appearance, can explain the higher scores among women (Eriksson et al., 2008). In another study, higher scores were associated with more sports activity (Varga et al., 2014). Segura-García et al. (2012) compared athletes with sedentary individuals and found that it was more common for athletes (e.g., individuals participating in judged sports, team sports, and fitness activities) to score higher. Independent predictors were a need to follow ritualized exercise patterns and involvement in professional sports.

### Empirical-holistic approach

This approach deals with an article that examines women's dietary behaviours. The article explores what women normally ate, the main ways they obtained food, how they chose the foods they ate, and how their diet had changed over time. Furthermore, the article focuses on general changes and trends regarding food within Western societies that might influence women's dietary behaviours. Women place an emphasis on *individual responsibility*, *decision-making*, *and confusion/anxiety regarding dietary health decisions*, which constitutes this category. Scholars have argued that these findings support the statement that Western societies have become orthorexic societies (Rangel, Dukeshire, & MacDonald, 2012).

### Rational-holistic approach

Nicolosi (2006) introduces and discusses the concept of *orthorexic society* that constitutes the included category within this approach. An orthorexic society is used as a “representation of an epoch-making condition” (p. 38) wherein contemporary Western societies are characterized by alimentary fears about ingesting the correct food. Food perfection and increased distance from industrialized food production practices represent the base and contradictions within an orthorexic society.

### Synthesizing examined categories sorted by philosophy of science approaches on three levels

**Figure 3:**
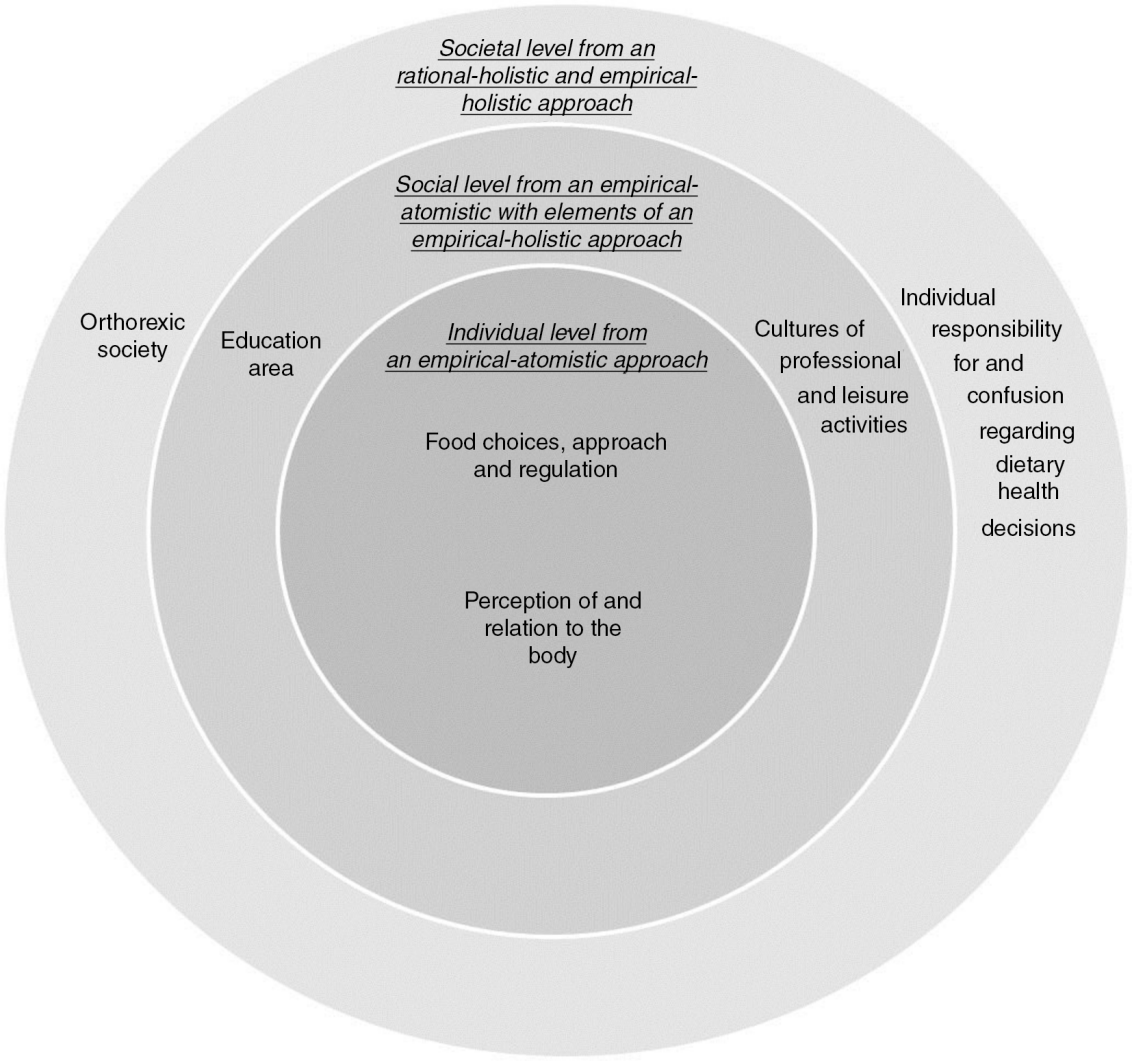
A model that synthesizes the categories that have been examined in relation to orthorexia, sorted by philosophy of science approaches at an individual, social, and societal level.

The multifaceted nature of the research demonstrates that orthorexia has been researched on individual, social, and societal levels using four different approaches (Figure 3). Even so, most of the articles corresponded to an individual level from an empirical-atomistic approach that focused on orthorexia as an individual issue. Sorting and synthesizing the research according to these different levels illustrate that individuals are influenced by multiple factors and systems on varying levels. Thus, the environment that surrounds individuals should be regarded as a series of nested levels; each outer level is inclusive of the inner ones (cf. levels within social-ecological model; Stokols, 1996). The first inner circle is called the *individual level* and covers the aspects that immediately influence the individual, such as the individual's behaviours, biology, and psychological and medical conditions. The next circle refers to the *social level*, which involves contextual and cultural spheres that influence the individual and in which the individual takes part. The third circle, the *societal level*, refers to wider sociocultural contexts in which individuals live, including societies and dominating contemporary health perspectives (cf. Stokols, 1996). Because it is not possible to present a clear picture of the findings, it is important to emphasize that the categories that are presented within the model are aspects that have been examined. Indeed, there is not necessarily consensus regarding how these aspects relate to orthorexia.

## Discussion

### Individual level from an empirical-atomistic approach

The individual level includes categories that are presented within the empirical-atomistic approach. In addition, it is important to state that the studies use different unreliable instruments, which means that the results must be interpreted with care and that it is not possible to draw general conclusions. Moreover, because different instruments have been used it is difficult to compare the results across different studies (see Table I).

Thus far, most of the articles have focused on the individual level of the empirical-atomistic approach, and thus they have been representing orthorexia as an individual issue, i.e., as a sum of the parts (mostly individual variables). By mainly focusing on this level, the studies have merely reproduced the idea of healthism, which means that the blame for health problems and the burden of their solutions are placed on the individual (Crawford, 2006; Kirk & Colquhoun, 1989). From a healthiest perspective, the patterns of behaviour within the inner circle should not only be regarded as being on the level of the individual but also as constrained behaviours or self-surveillance in response to the normative social pressures of healthism (cf. Crawford, 2004) that subsequently have been transformed into unhealthy or even harmful or detrimental behaviours.

On this level, because ORTO-15 is used as a diagnostic tool to determine the prevalence of orthorexia, the individual will take the blame as long as orthorexia is described in individual terms rather than as consequences of particular societies. A similar critique has been launched against the medical model that underpins existing research on eating disorders (Busanich & McGannon, 2010). Instead, these disorders should be described as multicausal illnesses (Papathomas, 2014) with psychological and biological explanations as well as social and cultural influences (Polivy & Herman, 2002). The aspects on the individual level are of significance but do not offer a full explanation because orthorexia occurs in the context of societal factors that shape behaviours.

The categories *food choices* and *approach*, *and regulation* (including dieting and weight regulation) that has been examined on the individual-level reflect healthism. This category refers to eating specific foods that are considered to be healthy, along with strict planning. These findings, including exercise, reflect healthism because healthy eating and exercise are depicted as means to achieving healthiest health (Kirk & Colquhoun, 1989; Wright et al., 2006). *Food choices* and *approach and regulation* also reflect healthism because these aspects can be seen as examples of taking responsibility for protecting one's self against health threats such as the obesity epidemic.

Furthermore, the category *perception of and relation to the body* can be tied to moral obligations that are elements within healthism and is linked to body size and shape (Crawford, 1980). A fit body represents health and is a symbol of good living; an obese body is regarded as lazy, emotionally weak, and unattractive (Crawford, 1987). *Perception of and relation to the body*, including body weight anxiety, can be seen as a response to these moral obligations. When these obligations value individuals on the basis of their body weight, people feel that it is important to possess the “correct” body weight. Indeed, individuals are expected to achieve health, to avoid risk factors, and to prevent ill health (Crawford, 1980), which includes not being overweight. For example, individuals are expected to protect themselves from the “obesity epidemic” (Gard & Wright, 2005). As long as articles focus on individual aspects, they will reproduce the idea that health problems are an individual's responsibility (Crawford, 2006; Kirk & Colquhoun, 1989).

### Social level from an empirical-atomistic with elements of an empirical-holistic approach

This level combines the categories *education area* and *culture of professional and leisure activities* that are presented within the empirical-atomistic approach with elements of the empirical-holistic approach. These categories have partly been examined as potential risk groups and have, to some extent, been discussed in relation to the high scores on orthorexia instruments (i.e., these studies do not exclusively focus on the individual level). In Table I, one can see that participants within other cultural contexts (e.g., medical doctors and dieticians; see the column “participants” for more examples) have been included in the articles presented on other levels, but these participants have not been discussed to the same extent as those participants who are presented on this social level.

The category *culture of professional and leisure activities* highlights the correlation of sports and exercise with higher scores on orthorexia instruments (e.g., Varga et al., 2014). However, it is still unclear what role sports and exercise plays in relation to orthorexia; for instance, whether fitness participants are a risk group (Eriksson et al., 2008), whether exercise or sport involvements are associated with orthorexia (Segura-García et al., 2012), and whether exercise should be considered to be a symptom of orthorexia (Varga et al., 2014). We must question, then, whether orthorexia has extended or is being extended to mean or include other behaviours than those originally noted by Bratman (1997; Bratman & Knight, 2000).

However, when elite sports and exercise participation are related to orthorexia, it is important to question the definition of orthorexia because the norms and values inside and outside the sports setting differ (cf., as cited in Tan, Bloodworth, Mcnamee, & Hewitt, 2014). Within formal sports settings, strict food and exercise regulations are common (Dale & Landers, 1999; Sundgot-Borgen & Torstveit, 2010). Previous research has shown that among elite athletes in combat sports, these behaviours are described as part of the athletes’ identities as well as the sports culture (Pettersson, 2013). This culture is partially highlighted in an article by Segura-García et al. (2012) that deal with orthorexia. The article proposes that rigid dietary habits may be regarded as standard practices among performance-oriented athletes and may also be considered incorrect among physically active individuals who score high on orthorexia instruments. This disparity demonstrates the importance of context in determining what is considered healthy, what is considered idealized practice, and what are considered “orthorexic behaviours.” Clarifying values can discourage detrimental behaviour from becoming normalized within the sports context and discourage appropriate behaviour for professional athletes from becoming pathologized. A similar pattern is also evident among ashtanga yoga practitioners, who score high on the ORTO-15. Scholars recognize that a healthy diet is a standard component of yoga practice and is, therefore, not necessarily a problem (Valera et al., 2014).

The *culture of professional and leisure activities* category involves exercise and fitness participation, which belongs to the empirical-atomistic approach with elements of the empirical-holistic approach. We decided to place exercise and fitness findings on a social level because they discuss the cultural context to some extent. It is fruitful to discuss exercising at a fitness centre at this level because it is not merely an individually driven action. Indeed, healthism has been pointed out to be a dominant ideology within different settings (e.g., health promotion efforts and popular media) in developed Western societies (Lee & Macdonald, 2010) and the fitness centre culture can perhaps be identified as one of these healthiest settings. For instance, exercising, dieting, focusing on body shape, and pursuing good health are markers of a fitness culture (e.g., cf. Andreasson & Johansson, 2013). Thus, the different settings that are presented on the social level highlight the meaning of contextualization regarding orthorexia within different practices.

### The societal level from an empirical-holistic and rational-holistic approach

This level includes the categories *individual responsibility*, *decision-making*, *and confusion/anxiety regarding dietary health decision* and *orthorexic society* that are presented within the empirical-holistic approach and the rational-holistic approach. Two studies (Nicolosi, 2006; Rangel et al., 2012) were conducted from a holistic approach in which the authors introduced and used the theoretical concept of an *orthorexic society*. These two studies’ positions undermine orthorexia as an individual pathology. As a result, these studies currently contribute to the holistic approach. Nicolosi (2006) began by introducing the concept of an orthorexic society, and Rangel et al. (2012) used this concept in an empirical study and argued that they could find empirical evidence for the existence of such societies. Both studies’ conceptualizations extended orthorexia to a representation of an “epoch-making” condition because orthorexia occurs in societies with food perfection, which involves food organizing, research, and selection (Nicolosi, 2006). Despite the introduction of this concept, there is still a limited contextualization of orthorexia because of the small body of knowledge on a social level conducted from holistic approaches. Now that these two studies have introduced an orthorexic society, future studies can consider the critical aspects and consequences regarding food perfection. For example, this society's focus on food is consistent with Bratman's description of orthorexia, which involves a focus on an unhealthy obsession with eating healthy foods (cf. Bratman & Knight, 2000). Because orthorexic society deals with problematic aspects regarding food in contemporary Western societies, Bratman's description of orthorexia is being considered and in some way is also problematized on a societal level. Sports and exercise are not discussed in Bratman's (1997) description of orthorexia and have not been connected to orthorexic society. We propose, however, that adding the theoretical concept of healthism will be generative for researchers to consider sports and exercise.

Finally, our findings indicate that the research of orthorexia is disparate, which reinforces that it is still being established. Orthorexia can be regarded as a new and emerging topic that does not have its own code of diagnosis or clear descriptions, rendering it challenging to research. The model (Figure 3) may serve as a starting point in developing a holistic view of and approach to researching orthorexia. We recommend that orthorexia be viewed as qualitatively larger than the sum of its parts. Future research can, for instance, add levels within the model and examine what aspects within the different levels that are linked to each other and how these levels and aspects relate to the individual and influence unhealthy behaviours.

### Critical discussion

The majority of the articles adhered to the empirical-atomistic approach (Figure 2) conducted on an individual level (Figure 3), which means that the main perspectives used to study orthorexia thus far have been medical and psychological (i.e., individual pathologies) and are based on an empirical-atomistic philosophy of science assumption. Thus, the literature lacks examinations that are based on other philosophy of science assumptions and predominantly conceptualizes orthorexia as an individual issue (cf. Rangel et al., 2012). This approach builds on atomism, which refers to the world as the sum of its parts (Gunnarsson, 2014). For instance, orthorexia is explored and understood as the sum of related variables that are investigated within the research. Knowledge within this approach is important but cannot itself represent orthorexia because orthorexia occurs in a context of societal factors, including contemporary healthism.

Even though most of the studies applied the empirical-atomistic approach on an individual level, it is not possible to present a clear picture of orthorexia based on the present literature (cf. Ramacciotti et al., 2011) because of (a) the small body of knowledge that is characterized by inconsistent results, (b) the varying use of different orthorexia instruments (Ramacciotti et al., 2011), and (c) the fact that the participants derive from a wide range of professions and countries. There are also deficiencies with the instruments that have been used. The BOT measure has been neither validated nor psychometrically evaluated (Alvarenga et al., 2012). When ORTO-15 was proposed for the first time, neither a factor nor an internal consistency analysis was conducted on it (Alvarenga et al., 2012; Donini, Marsili, Graziani, Imbriale, & Cannella, 2005). Nevertheless, both these measurements have been used in a variety of studies, particularly the ORTO-15 (see Table I), which makes it difficult to compare the results of different studies. There may also be limitations to the instruments’ sensitivities or cut-offs given that high scores on orthorexia instruments were prevalent in some studies (exceeding 80%). Alvarenga et al. (2012) examined the high scores on the orthorexia instruments and argued that it might not be appropriate to have a cut-off in an instrument that aims to measure a non-diagnosis. These authors also discussed whether the questions included in the ORTO-15 are appropriate for evaluating “orthorexic tendencies.” Some of the questions are difficult to classify as dysfunctional signals because individuals who eat healthfully could also score high on some of the questions. Nevertheless, several articles implicitly use instruments with a cut-off as diagnostic tools for orthorexia. In some of the articles, scholars explain that orthorexia is not a diagnosis but at the same time handle orthorexia as a diagnosis by presenting its prevalence. Instead, it would perhaps be more appropriate to state that individuals score high on the instrument. An additional aspect is that the instruments do not consider temporal evolution. For instance, an individual could score high on the ORTO-15 because of a period of dieting that is transient. Indeed, when diagnostic criteria are proposed for orthorexia, Varga et al. (2013) recommend that the behaviours have persisted for 6 months or more.

An unresolved question within a social level from an empirical-atomistic with elements of the empirical-holistic approach is whether orthorexia instruments are suitable for all kinds of contexts. Scholars argue that athletes might be a risk group since they score higher on the ORTO-15 compared with, for example, the general population in Italy (Segura-García et al., 2012). Higher scores on orthorexia instruments among individuals engaging in sports activities have been found (Varga et al., 2014). Scholars claim that individuals engaging in professional sports might be at higher risk because they undergo rigid dietary regulations. For instance, proper nutrition is considered to be an essential aspect of optimal performance within sports (Segura-García et al., 2012). Therefore, we ask whether the instruments examine unhealthy and even detrimental behaviours or examine a specific culture in different contexts. For instance, ashtanga yoga practitioners also score high on ORTO-15 because emphasis on a healthy diet is part of the culture of yoga practice. This behaviour does not necessarily influence individuals’ lives negatively (Valera et al., 2014). Therefore, the questions on the ORTO-15 should be assessed for cultural appropriateness because they might not always be appropriate for examining unhealthy or detrimental behaviours. Thus, orthorexia and various sociocultural settings need to be considered and problematized to prevent detrimental behaviour from becoming normalized and the participants from becoming pathologized.

### Method discussion

One limitation of this study is the keywords that were used: we searched the articles using three words. For example, the keywords would not have returned studies that dealt explicitly with restrained eating. Nevertheless, we did consider our choice of keywords. Had other keywords been included, we would have been implying that they were part of orthorexia. Indeed, the relationship between orthorexia and exercise is still unclear. Studies that have other keywords do not explicitly contribute to the establishment of the concept of orthorexia.

### Research implications

In general, more research dealing with orthorexia is required because of all the inconsistent results and deficiencies with the methods that have been used thus far. In particular, research using empirical-holistic approaches that involve qualitative studies is necessary to examine individuals’ perceptions and experiences of this condition. Future studies should also emphasize a bottom-up perspective and ensure that the voices of individuals are heard. Research using rational-holistic and empirical-holistic approaches, on the other hand, can explore orthorexia from a contemporary social perspective of health. Such a holistic approach could contextualize orthorexia by, for instance, conducting dialogues with individuals who experience orthorexia, thereby making meaning of the context. The holistic approach could also be a starting point in developing instruments for orthorexia. However, the authors acknowledge that recruitment of participants would be challenging because orthorexia does not have its own code of diagnosis and is not clearly defined. Finally, the potential relationship among sport, exercise, and orthorexia should be additionally explored, examined, and discussed.

## Conclusion

This integrative review has demonstrated that orthorexia has been researched on the individual, social, and societal levels using four different philosophy of science approaches (i.e., empirical-atomistic, empirical-atomistic with elements of empirical-holistic, empirical-holistic, and rational-holistic) not previously identified. The majority of articles adhered to the empirical-atomistic approach, which indicates the limited contextualization of this condition and thus contributes to depicting orthorexia as an individual issue. Healthism was used to discuss and contextualize research on orthorexia. The review also showed that sports and exercise have been examined in relation to orthorexia within research but that their interrelationships remain unclear. The findings of this integrative review can generate further inquiries into orthorexia; several epistemological questions and implications for orthorexia have already surfaced as a result of this review. This review indicates a need for (a) more empirical-holistic research that applies interpretive qualitative methods and uses a social perspective of health (e.g., healthism) and (b) examining the role of sports and exercise in relation to orthorexia that takes into account the problematizing of “orthorexic behaviours” within the sports context.

## Notes

1 The majority of the scientific articles examine the prevalence of orthorexia. High scores on orthorexia instruments are described as a tendency for orthorexia or as orthorexia. We prefer to describe that individuals score high on an orthorexia instrument as orthorexia is not a diagnosis. Even so, when the scientific articles are summarized within the table, the scholars’ aims are not rewritten into high scores.

2 In general, scholars mostly describe high orthorexia scores as a higher tendency for orthorexia or as orthorexia. However, we have chosen to describe these tendencies simply as higher scores on orthorexia instruments.
